# The Possibility of Changing the Wettability of Material Surface by Adjusting Gravity

**DOI:** 10.34133/2020/2640834

**Published:** 2020-01-27

**Authors:** Yong-Ming Liu, Zi-Qing Wu, Sheng Bao, Wei-Hong Guo, Da-Wei Li, Jin He, Xiang-Bin Zeng, Lin-Jun Huang, Qin-Qin Lu, Yun-Zhu Guo, Rui-Qing Chen, Ya-Jing Ye, Chen-Yan Zhang, Xu-Dong Deng, Da-Chuan Yin

**Affiliations:** ^1^Key Lab of Space Bioscience & Biotechnology, School of Life Sciences, Northwestern Polytechnical University, Xi'an, 710072 Shaanxi, China; ^2^School of Bioengineering, Sichuan University of Science and Engineering, Zigong, 643000 Sichuan, China; ^3^Shenzhen Research Institute of Northwestern Polytechnical University, Shenzhen, 518057 Guangdong, China

## Abstract

The contact angle, as a vital measured parameter of wettability of material surface, has long been in dispute whether it is affected by gravity. Herein, we measured the advancing and receding contact angles on extremely low contact angle hysteresis surfaces under different gravities (1-8G) and found that both of them decrease with the increase of the gravity. The underlying mechanism is revealed to be the contact angle hysteresis and the deformation of the liquid-vapor interface away from the solid surface caused by gradient distribution of the hydrostatic pressure. The real contact angle is not affected by gravity and cannot measured by an optical method. The measured apparent contact angles are angles of inclination of the liquid-vapor interface away from the solid surface. Furthermore, a new equation is proposed based on the balance of forces acting on the three-phase contact region, which quantitatively reveals the relation of the apparent contact angle with the interfacial tensions and gravity. This finding can provide new horizons for solving the debate on whether gravity affects the contact angle and may be useful for the accurate measurement of the contact angle and the development of a new contact angle measurement system.

## 1. Introduction

Wetting is one of the basic characteristics of solid surfaces. It is very important for processes like adhesion [1], self-cleaning [2], fluid drag reduction [3], heterogeneous nucleation [4], and the formation of coffee rings [5]. Therefore, it has attracted immense interest in a large diversity of research fields ranging from physical, biological, and environmental sciences. Owing to its complexity, wetting and the parameter used to measure it, the contact angle, have been investigated for many years [6–27]. Currently, thousands of papers are published annually about the topic. However, there are still fundamental questions to be answered. The relationship between the wetting and gravity is one of them.

In 1805, Young pointed out that every solid-liquid pair has an “appropriate angle of contact” [6]. This “appropriate angle of contact” is called Young's contact angle. According to his description, a famous equation named Young's equation can be written as

(1) $$\begin{array}{c} \gamma_{\text{lv}}\cos\theta_{\text{Y}}=\gamma_{\text{sv}}-\gamma_{\text{sl}}, \end{array}$$

where *γ*_lv_ is the liquid-vapor interfacial tension, *γ*_sv_ is the solid-vapor interfacial tension, *γ*_sl_ is the solid-liquid interfacial tension, and *θ*_Y_ is Young's contact angle for a drop on a solid.

Because the disjoining pressure, resulted from the intermolecular interaction, makes the structure of three-phase contact line complicated, Benner et al. [28] referred to Young's equation not being valid, and the alternative equations for contact angle were derived by other researchers [29–32] based on various intermolecular force models. This issue was resolved by Keller and Merchant [33], and a precise mathematical definition for the contact angle was proposed: a boundary condition to the Young-Laplace equation where the film thickness is 0. Physically, as addressed by de Gennes [34], Young's contact angle is a measurable macroscopic contact angle which is on a scale above that of long-ranged intermolecular forces [34]. At present, it is considered that Young's equation describes the relationship between macroscopic, measurable, thermodynamic variables, and the contact angle. And the interfacial tensions refer to the constant, interfacial Gibbs free energies far from the contact line. In Young's equation, gravity is not included as a variable.

Some researchers [8–14] also derived the same Young's equation based on the thermodynamics of wetting and pointed out that the contact angle depends only on the physical and chemical properties of the solid, liquid, and vapor accordingly and is not affected by gravity. Gravity only affects the shape of the drop [8–14]. Recently, Bormashenko imposing the transversality conditions on the variational problem of wetting also demonstrates that gravity does not influence equilibrium contact angles [35–37]. However, many experimental observations [15–27] under some gravities (≤2G) differed from these theoretical conclusions. This discrepancy becomes an important issue, especially in the space era, when interfacial phenomena frequently draw more attention because they are dominant events in microgravity and much different from those observed on Earth. Extensive studies on wetting and the contact angle are beneficial for clarifying this issue.

It is generally believed that Young's contact angle represents the contact angle of the liquid on an ideal surface, which refers to a rigid, smooth, chemically homogeneous, and inert surface. On an ideal surface, the system has a single and unique contact angle. However, for a real solid surface and a liquid, many contact angles can be measured since the system has many metastable equilibrium states, and each metastable equilibrium state corresponds to one contact angle [38]. Among these contact angles, the lowest metastable contact angle is the receding contact angle, and the highest one is the advancing contact angle [38]. They can be measured by receding and advancing liquid on a solid surface [7, 38, 39]. And the difference between advancing and receding contact angles is called contact angle hysteresis. Nearly all real solid surfaces exhibit contact angle hysteresis [7, 39, 40]. Only a few smooth, chemically homogeneous, and inert real surfaces possess very low contact angle hysteresis [41, 42]. They are the ones that most closely approach an ideal surface. The contact angles on these low contact angle hysteresis surfaces are very close to Young's contact angle. Previous experimental studies [15–27, 43] at different gravities used ordinary surfaces. Thus, the results of the effect of gravity on the contact angle may be caused by contact angle hysteresis. To rule out this possibility, it is necessary to systematically study the relationship between the contact angle and gravity using surfaces with low contact angle hysteresis.

In addition to the requirement for using low contact angle hysteresis surfaces, clarification of the relationship between the contact angle and gravity needs to consider the drop size.

Even on ideal surfaces, the contact angle is affected by the drop volume increases, due to line tension *σ* [9, 44]. Equation (2) is the line tension-modified Young's equation:

(2) $$\begin{array}{c} \gamma_{\text{sv}}=\gamma_{\text{lv}}\cdot \cos\theta_{\text{Y}}+\gamma_{\text{sl}}+\frac{\sigma }{R}, \end{array}$$

where *R* is the three-phase contact radius.

According to Equation (2), when the drop is large enough, the effect of the line tension *σ* can be ignored [7, 38]. Furthermore, in order for the measurement and interpretation to be meaningful, the drop must be sufficiently large compared with the size scale of heterogeneity that ensures the drop base is axisymmetric [45]. Therefore, reproducible and reliable measurement of the contact angle shall be carried out using large drops. In the literatures, it has been reported that the radius of the sessile drop should be larger than 2.5-3.5 mm [46] or even larger than 3.0-5.0 mm [7], especially for chemically and morphologically heterogeneous surfaces. In previous experimental measurements of the contact angle under different gravities, large drops were rarely used.

In general, the sessile droplet on an inclined plate (real surface, not idea surface) will be deformed due to the pinning of the contact line and gravity, and the contact angle will be changed by gravity [47–51]. This issue was widely investigated by researchers [47–51]. The tilt plate method is also used to measure the advancing and receding contact angle. However, they are related to the weight of the drop. According to literature report, the advancing and receding contact angles obtained by the tilt plate method are not consistent with that obtained by the sessile drop [38, 45]. Thus, the sessile drop method is employed in this study.

In this study, the advancing and receding contact angles of large drops on solid surfaces with extremely low contact angle hysteresis under a wide range of gravities (1-8 G) were measured for the first time. The large range of hypergravities (1-8 G) were generated by a long-arm (*d* = 6 m) centrifuge which was specially designed and developed for the accurate measurement of apparent contact angles via a remote control. The dynamic process of gravity affecting the apparent contact angle was analyzed by solving the augmented Young-Laplace equation. The relationship between the contact angle measured by the optical method and the real contact angle was discussed. And a new equation describing the relationship between gravity and the apparent contact angle was presented. The discovery can provide new horizons for solving the debate on whether gravity affects contact angle and may be useful for the accurate measurement of the contact angle and the development of a new contact angle measurement system.

## 2. Results

### 2.1. Long-Arm Centrifuge and Contact Angle Measurement Unit

**Figure 1:**
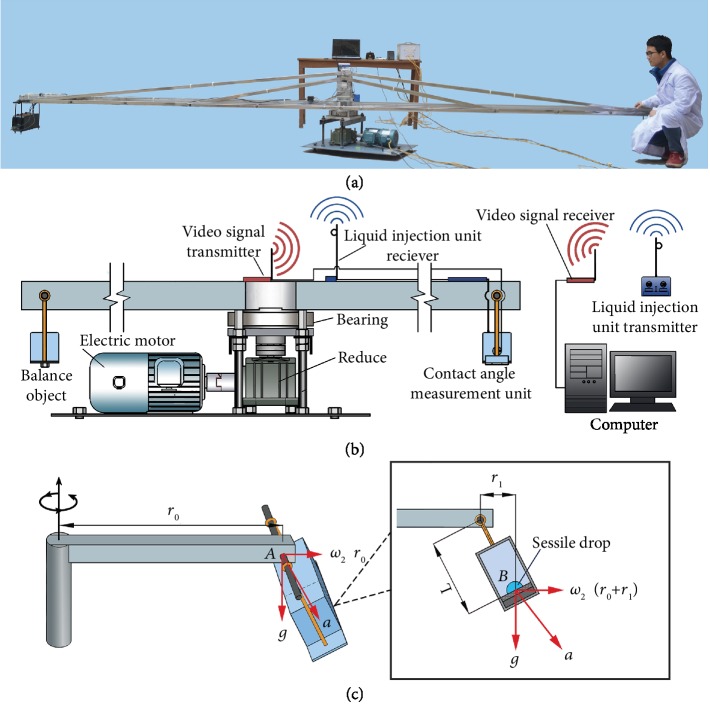
Long-arm centrifuge and contact angle measurement unit. (a) Photograph of the long-arm centrifuge and contact angle measurement unit. (b) Diagram of the long-arm centrifuge and contact angle measurement unit. (c) Diagram of centrifugation force. In a gravitational field, the acceleration of an object with the vector sum of the centrifugal and gravity forces at point *A* is $\sqrt{g^{2}+{\left(\omega^{2}r_{0}\right)}^{2}}$, and at point *B*, it is $\sqrt{g^{2}+{\left[\omega^{2}\left(r_{0}+{\text{r}}_{1}\right)\right]}^{2}}$. Owing to *L*<<*r*_0_, $\sqrt{g^{2}+{\left[\omega^{2}\left(r_{0}+{\text{r}}_{1}\right)\right]}^{2}}\approx \sqrt{g^{2}+{\left(\omega^{2}r_{0}\right)}^{2}}$. The centrifugal acceleration gradient of the centrifuge is ~0.0028 G/mm.

The contact angles of liquids on solid surfaces were measured under different gravities generated by a home-made specially designed long-arm (*d* = 6 m) centrifuge (Figures 1(a) and 1(b)). The maximum rotation rate of this centrifuge is 65 RPM. It can provide a stable and long durable gravity level of 1-8 G. The contact between the liquid and solid surface takes place at point *B* which is inside a sealed box in the contact angle measurement unit (Figure 1(c)). The contact angle measurement unit (Figure 1) hangs on one end of the long-arm, while an object of the same weight hangs on the other end of the long-arm for balance (Figure 1(b)). The liquid can be injected onto or withdrawn from the solid surface through a syringe via a remote liquid control unit (Figure 1(b)). A CCD camera equipped with a low distortion telecentric lens (resolution: 22~37 lp/mm) is used to capture the contact process between the liquid and solid surface, and a video of this process can be transmitted by a wireless video transmission system (Figure 1(b)). From the video, the images of the sessile drops can be captured, and the contact angle can be determined from the images using the DropSnake program [52].

### 2.2. Contact Angles at Different Gravities

**Figure 2:**
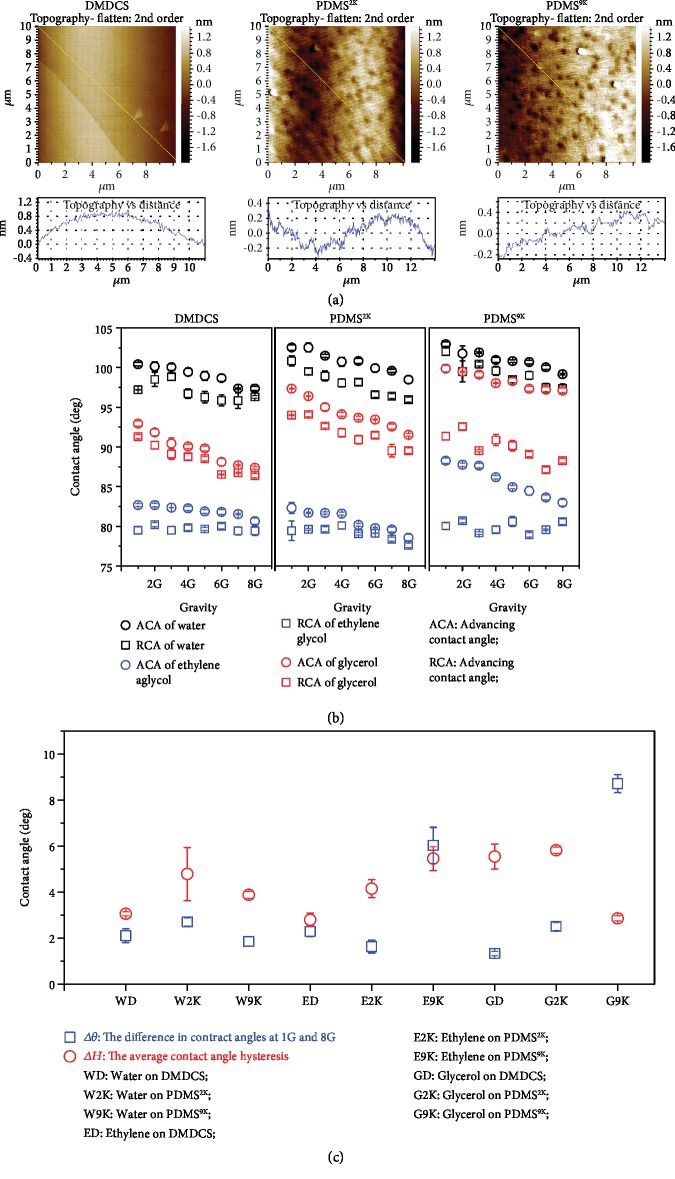
Apparent contact angles versus gravity. (a) AFM images of the sample solid surfaces and their section analyses. The analyses show that the surfaces are very smooth, with roughness less than 1 nm. (b) The contact angle on solid surfaces at different gravities. The contact angles show the same tendency to decrease with increasing gravity. The contact angle hysteresis was smaller than 3°, except for the cases of ethylene glycol and glycerol on PDMS^9K^. (c) Comparison of Δ*θ* (the difference in contract angles at 1 G and 8 G) and Δ*H* (the average contact angle hysteresis). Independent *t*-test was applied, *n* = 3, *P* < 0.05, *P* < 0.001, *P* < 0.001, *P* < 0.05, *P* < 0.001, and *P* < 0.001, for water on DMDCS, water on PDMS^9K^, ethylene glycol on PDMS^2K^, glycerol on DMDCS, glycerol on PDMS^2K^, and glycerol on PDMS^9K^, respectively. Five out of the nine solid-liquid contact systems showed significant larger Δ*θ* than Δ*H*, three showed comparable results. The experimental results confirmed that the decrease in the contact angle upon increasing gravity is caused by gravity, not only by the contact angle hysteresis.

In this work, silicon wafers were treated using DMDCS (dimethyldichlorosilane), PDMS^2K^ (trimethylsilyl-terminated linear poly (dimethylsiloxane), MW 2,000), and PDMS^9K^ (trimethylsilyl-terminated linear poly (dimethylsiloxane), MW 9,000) to obtain smooth surfaces with low contact angle hysteresis. Their morphologies were studied by AFM (Atomic Force Microscope) (Figure 2(a)). It can be seen that the roughness of all surfaces is ~1 nm. Compared with the DMDCS, PDMS^2K^ and PDMS^9K^ have many small hills, and those of the PDMS^9K^ are rougher than those of the PDMS^2K^. The low-rate dynamic contact angles of water, ethylene glycol, and glycerol on these surfaces were measured under 1-8 G. [Supplementary-material supplementary-material-1] shows the measurement results of the contact angle of water on DMDCS at different gravities. The results show that the DMDCS was indeed low contact angle hysteresis surface. Most of the other surfaces also exhibited the same properties. Figure 2(b) shows the contact angles of different liquids on different solid surfaces at 1-8 G. It can be seen that the contact angle hysteresis was very low (<3°) in most cases except for ethylene glycol and glycerol on PDMS^9K^. Generally, surface roughness and chemical heterogeneity will lead to pinning of the three-phase contact line, which subsequently results in contact angle hysteresis [35, 53]. For the PDMS^9K^, the pinning effect of these small hills on the three-phase contact line may be the reason for the relatively larger contact angle hysteresis.

From Figure 2(b), it can also be seen that the apparent contact angle decreased as gravity increased, especially for the advancing contact angle. Although low contact angle hysteresis surfaces were used in this study, the contact angle hysteresis still exists. Thus, the decrease of the apparent contact angle with the increase of gravity may be related to contact angle hysteresis.

For a drop on a real surface, the wetting state is generally in a metastable equilibrium state, and the most stable equilibrium state is difficult to achieve because many energy barriers need to be overcome [38]. The advancing and receding contact angles can be easily measured because of the low energy barrier [38]. Generally, additional energy can overcome the energy barrier and make the wetting state reach a more stable equilibrium state [38, 45]. The direct result is that the additional energy decreases the advancing contact angle and increases the receding contact angle [38]. The increasing gravity may provide additional energy to make wetting reach a more stable state, so that the advancing and receding contact angles are different under different gravities. However, upon checking the data in Figure 2(b) more carefully, we found that all receding contact angles do not increase with the increase of gravity as predicted by theory except receding contact angles of ethylene glycol on DMDCS and PDMS^9K^. In addition, by comparing Δ*θ* (the difference in the contact angles at 1 G and 8 G) and Δ*H* (the average contact angle hysteresis) (Figure 2(c)), we also found that in six of nine liquid-solid contact systems, Δ*θ* was greater than Δ*H*. This means that the apparent contact angle decrease relative to the increasing gravity was not only caused by contact angle hysteresis.

### 2.3. Hydrostatic Pressure at Different Gravities

As showed in Figures 2(b) and 2(c), the apparent contact angles are affected by gravity. The direct consequence of gravity for a drop is the presence of the hydrostatic pressure, which means that the apparent contact angle under gravity is related to the hydrostatic pressure.

**Figure 3:**
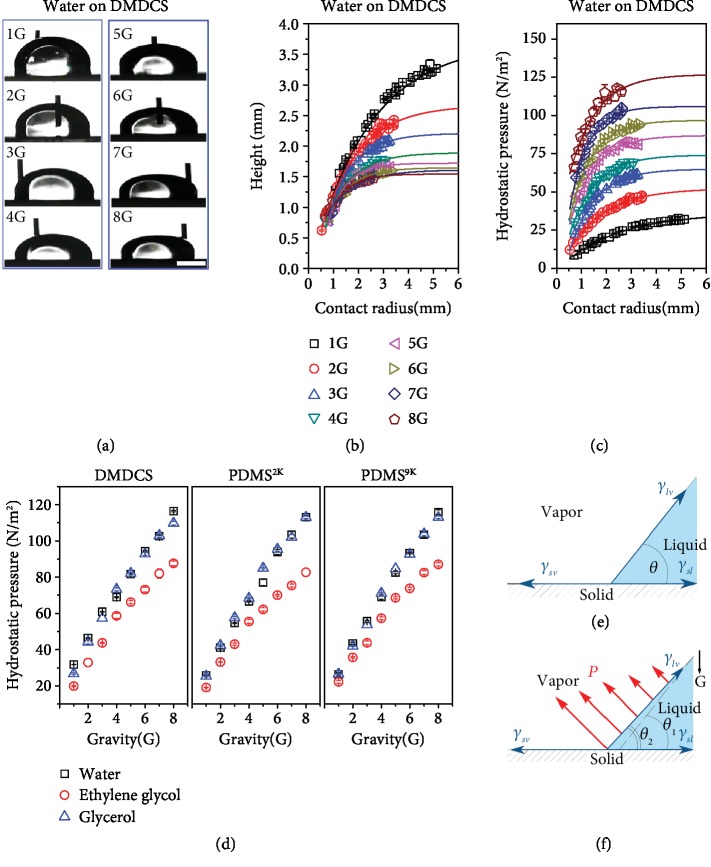
Hydrostatic pressure at different gravities. (a) Sessile water drop on DMDCS at different gravities (*R* = 2.5 mm). At higher gravity, the drop deforms and its height decreases more obviously. (b) Height versus the three-phase contact radius for sessile water drops on DMDCS surface under 1-8 G gravity. (c) Hydrostatic pressure at the three-phase contact line versus the three-phase contact radius for sessile water drops on a DMDCS surface under 1-8 G gravity. (d) Hydrostatic pressure at the three-phase contact line versus the different gravities. (e) Conventional analysis of the equilibrium of forces near the three-phase contact line (gravity is not considered). (f) Equilibrium of forces near the three-phase contact line of a sessile drop (gravity is considered). The experimental measurement shows that although the height decreases, the hydrostatic pressure increases monotonously with gravity, indicating that hydrostatic pressure should not be neglected, especially when gravity is large. The qualitative analysis in (f) shows that gravity can decrease the contact angle.

Figure 3(a) shows example images of the sessile drop (water on DMDCS, *R* = 2.5 mm) under gravities ranging from 1 to 8 G. It can be seen that the height *h* of the drops decreased upon increasing gravity (Figure 3(b)). However, the hydrostatic pressure *ρ*gh at the three-phase contact line was increasing during the increase of gravity *g* (Figure 3(c)). This result means that the effect of gravity on the hydrostatic pressure is more significant than that of the drop height. The results of all hydrostatic pressure cases investigated in this research are summarized in Figure 3(d), which show that the hydrostatic pressure does increase with increasing gravity, despite the height of the drop decreasing with increasing gravity. However, how does the hydrostatic pressure affects the apparent contact angle?

In the conventional analysis of the equilibrium of forces near the three-phase contact line, Young's equation was obtained [6] (Figure 3(e)), and the hydrostatic pressure was not considered. In gravitational field, the diagram of the hydrostatic pressure (red arrows) acting on the liquid-vapor interface of the drop of the three-phase contact region is shown in Figure 3(f). The hydrostatic pressure increases from 0 to *ρ*gh from the top to the bottom of the drop. As shown in Figure 3(f), the drop will be deformed due to the hydrostatic pressure, leading to a smaller contact angle as compared with that without considering gravity. With increasing gravity, the hydrostatic pressure will increase, so that the deformation of the drop will be more significant, resulting in a smaller contact angle (Figure 3(d)).

However, theoretically, the contact angle has nothing to do with gravity, whether the disjoining pressure is ignored (such as derivation of Young's equation by Bormashenko [35, 36]) or considered (for an example, the classical work by Starov and Velarde [37]). This is in contradiction with our experimental results. The possible reason is that the contact angles we measured are the apparent contact angles, not the mathematically defined contact angles. They are on the liquid-vapor interface and away from the solid surface due to the low resolution of the measurement system. If the resolution of the measurement system is high enough, we will see the real three-phase contact region. In this region, viscous resistance, resulted from the intermolecular interaction, is very high. Compared with the disjoining pressure, ~10^6^ N/m^2^, the hydrostatic pressure (~10^2^ N/m^2^ under 8G) is much smaller, and it is impossible to deform the liquid-vapor interface in the three-phase contact region. The deformation of liquid-vapor interface caused by hydrostatic pressure can only occur in the area controlled by capillary action far away from the solid surface and the three-phase contact region. The situation shown in Figure 3(d) is just a macrosituation. In fact, the measured apparent contact angles are the angles of inclination of a certain position on the liquid-vapor interface. In order to confirm this point, it is necessary to study the relationship between droplet profile and inclination angle under different gravities.

### 2.4. Drop Profile, Angle of Inclination of Liquid-Vapor Interface, and Contact Angle under Different Gravities

**Figure 4:**
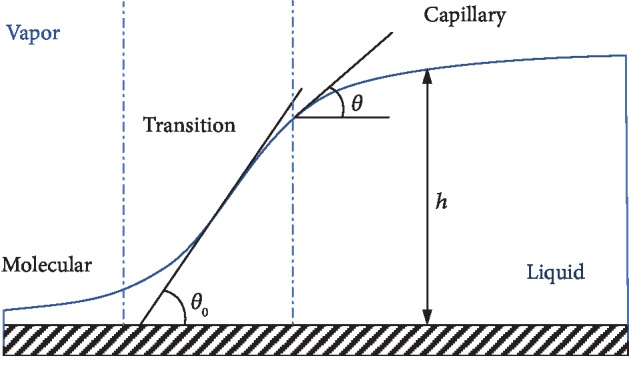
Interface shape in the vicinity of the contact line. There are three regions: capillary region, molecular region, and transition region. *θ*: angle of inclination of liquid-vapor interface; *θ*_0_: contact angle, where the surface forces are neglected and *h* = 0.

In this part, we use the method of Diaz et al. [31] to deduce the relationship between drop profile and inclination angle of liquid-vapor interface.  Figure 4 shows the 2D profile of a liquid-vapor interface shape in the vicinity of the contact line. As shown in Figure 4, there are three regions—molecular, transition, and capillary regions. The molecular region is dominated by the disjoining pressure and spatially varying interfacial free energies resulted from the molecular interaction; the capillary region is dominated by the capillarity and gravity; and in the transition region, the disjoining pressure competes with the hydrostatic pressure, and the surface tension is assumed constant. Within the molecular region, the equation for the shape of the liquid-vapor interface is the fully augmented Young-Laplace equation [31]:

(3) $$\begin{array}{c} g_{\text{lv}}\left(h,\theta \right)2H=-\prod \left(h,\theta \right)-\left(p_{l}-p_{v}\right), \end{array}$$

where *g*_lv_(*h*, *θ*) is liquid-vapor interfacial free energy, *h* is the film thickness, *θ* is the angle of inclination of the liquid-vapor interface, 2*H* is the curvature, *Π*(*h*, *θ*) is the disjoining pressure, *p*_l_ is the pressure in liquid, and *p*_v_ is the pressure in vapor. Above the molecular region, *g*_lv_(*h*, *θ*) becomes a constant, *γ*_lv_.

In Equation (3), 2*H* can be expressed by

(4) $$\begin{array}{c} 2H=‐\frac{d\cos\theta }{dh}. \end{array}$$

For convenience, considering only the contribution from Van der Waals force, the disjoining pressure can be expressed by

(5) $$\begin{array}{c} ‐\prod \left(h,\theta \right)=\frac{A_{LL}G\left(\theta \right)-A_{\text{SL}}}{6\pi h^{3}}, \end{array}$$

where *A* is Hamaker constants, *G*(*θ*) = (1/2) + (3/4)cos*θ* − (1/4)cos^3^*θ* [54].

By introducing a molecular film thickness, *h*_m_,

(6) $$\begin{array}{c} h_{m}=\frac{A_{\text{LL}}G\left(\theta_{0}\right)-A_{\text{SL}}}{6{\pi \gamma }_{\text{lv}}}, \end{array}$$

then,

(7) $$\begin{array}{c} \prod \left(h\right)=‐\gamma_{\text{lv}}\frac{h_{m}^{2}}{h^{3}}. \end{array}$$

For the liquid slice, the hydrostatic pressure at any point on the interface can be expressed by

(8) $$\begin{array}{c} p_{l}-p_{v}=\rho g\left(h_{e}-h\right), \end{array}$$

where *h*_*e*_, the equilibrium height of the drop, is a certain constant at a particular gravitational level; *h* is the height of any point on the liquid-vapor interface.

Outside the molecular region, an augmented Young-Laplace equation can be obtained by combining Equations (3), (4), (7), and (8)

(9) $$\begin{array}{c} ‐\frac{d\cos\theta }{dh}=\frac{h_{m}^{2}}{h^{3}}-\frac{h_{e}-h}{{\left(\kappa^{‐1}\right)}^{2}}, \end{array}$$

where $\kappa^{‐1}=\sqrt{\gamma_{\text{lv}}/\rho g}$ is the capillary length.

Integrating Equation (9) and imposing *h* = *h*_*e*_, *θ* = 0 yields the solution

(10) $$\begin{array}{c} \cos\theta =\frac{h_{m}^{2}}{2h^{2}}-\frac{h_{m}^{2}}{2h_{e}}+1-\frac{h_{e}^{2}}{2\cdot {\left(\kappa^{‐1}\right)}^{2}}+\frac{{hh}_{e}}{{\left(\kappa^{‐1}\right)}^{2}}-\frac{h^{2}}{2\cdot {\left(\kappa^{‐1}\right)}^{2}}. \end{array}$$

Without the disjoining pressure, Equation (10) becomes the Young-Laplace equation:

(11) $$\begin{array}{c} \cos\theta =1-\frac{h_{e}^{2}}{2\cdot {\left(\kappa^{‐1}\right)}^{2}}+\frac{{hh}_{e}}{{\left(\kappa^{‐1}\right)}^{2}}-\frac{h^{2}}{2\cdot {\left(\kappa^{‐1}\right)}^{2}}. \end{array}$$

**Figure 5:**
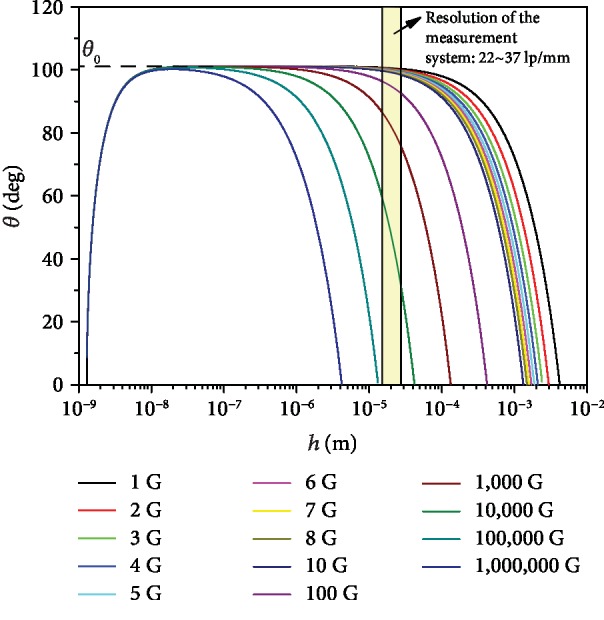
Variation of the water contact angle *θ* on DMDCS with the film thickness *h* under different gravities. The dashed line is the solution of the Young-Laplace equation (Equation (11)); the colored line is the solution of the augmented Young-Laplace equation (Equation (10)).

Figure 5(a) shows a variation of the angle *θ* of the water drop on DMDCS with the film thickness *h* under different gravity (*θ* is calculated by using Equation (10) and assuming *h*_*m*_ = 2 × 10^−9^ m). It can be seen that the transition region, where the film curvature is negligible, decreases with the increasing gravity. When gravity increases to 100,000 G, the linear transition region of the liquid-vapor interface begins to deform. However, the contact angle *θ*_0_ (*h* = 0 m) is independent of gravity. Only above the transition region, the angles of inclination of the liquid-vapor interface decrease with the increase of gravity.

In this study, the resolution of the measurement system is of 22~37 lp/mm. That is, the minimum size that the measurement system can identify is of 2.7 × 10^−5^~4.5 × 10^−5^ m. On this scale, our experimental results (Figure 2(b)) are in good agreement with the theoretical values (Figure 5). That means the measured apparent contact angles, *θ*_ma_, are angles of inclination of the liquid-vapor interface at a distance of 10^−4^~10^−5^ m from the solid surface. In other words, the measured apparent contact angles are not the real contact angles. The measured apparent contact angles depend on the resolution of the measurement system.

From Figure 5, we can also see that the lower the gravity is, or the higher the resolution of measurement system is, the closer the measured apparent contact angle is to the real contact angle. Therefore, it is suggested to measure the contact angle under a lower gravity environment or using a higher resolution measurement system.

In this section, for convenience, only the distribution of Van der Waals force to the disjoining pressure was considered. In fact, for water, other two components, electrostatic component and structural component, are also important components for the disjoining pressure. Unfortunately, there are no firm and precise theoretical equations for these two components [55]. The real shape of the three-phase contact region for water or aqueous solution under high gravity may be very complex.

### 2.5. Relationship between Apparent Contact Angle and Gravity

**Figure 6:**
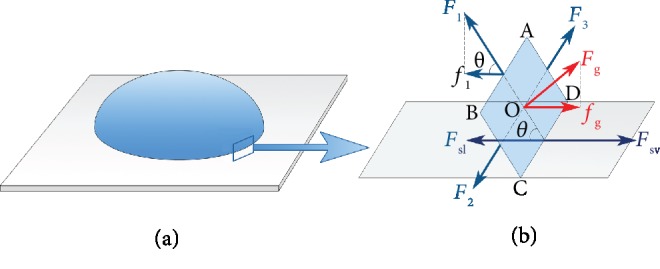
Equilibrium of forces near the three-phase contact line of sessile drop. (a) Diagram of a sessile drop. (b) Equilibrium of forces near the three-phase contact line. *F*_sl_ and *F*_sv_: solid-liquid and the solid-vapor interfacial tension forces; *F*_*g*_ and *f*_*g*_: force caused by the hydrostatic pressure and its horizontal component; *F*_1_, *F*_2_, and *F*_3_: liquid-vapor interfacial tension forces; *f*_1_*:* horizontal component of *F*_1_; *θ*: the apparent contact angle.

The contact angle is independent on gravity. However, the measured apparent contact angle, which is an angle of inclination of the liquid-vapor interface away from the solid surface, can be affected by gravity. It is related to the resolution of the measurement system. In order to clarify the relationship between the apparent contact angle and gravity, neglecting completely the disjoining pressure, we used the mechanical method of deriving the Young-Laplace equation [56], with consideration of the presence of hydrostatic pressure, liquid-vapor, solid-vapor, and solid-liquid interfacial tensions. And for this large drop, we consider a small rectangular section (ABCD, Figure 6) of the liquid-vapor interface at the three-phase contact line, where CD is a segment of the apparent three-phase contact line. The liquid-vapor interfacial tension forces pull the three edges of surface ABCD along the tangent direction perpendicular to the edges, and the solid-liquid and the solid-vapor interfacial tension forces *F*_sl_ and *F*_sv_ pull the side of CD along the horizontal direction. If the sessile drop is in gravity *g*, the surface ABCD will experience a hydrostatic pressure, resulting in a force *F*_*g*_ that is perpendicular to the surface. The sum of the forces in the horizontal direction must be zero (detail showed in Supplementary Materials). And a new equation can be written as below:

(12) $$\begin{array}{c} \gamma_{\text{sv}}=\gamma_{\text{lv}}\cdot \cos\theta +\gamma_{\text{sl}}-\rho {gh}_{e}h_{s}, \end{array}$$

where *h*_*e*_ is the height of the drop and *h*_*s*_,*BC* · sin*θ*, is an unknown length and is related to the resolution of the measurement system. When *h*_*s*_ equals to 0, the measured apparent contact angle will be equal to the real contact angle. When *h*_*s*_ is not equal to 0, the apparent contact angle is dependent on gravity *g*. The larger the *h*_*s*_ is, the more obvious is the influence of gravity on the contact angle.

From Equation (12), it can be seen that the dependence of the apparent contact angle on gravity depends on *h*_*s*_. Therefore, it is possible to use the calculated contact angle to fit the measured apparent contact angle by adjusting the value of the length *h*_*s*_. The schematic flowchart of the calculation process is shown in [Supplementary-material supplementary-material-1].  Table 1 shows the results of the value of *h*_*s*_. From Table 1, we can see that the scale of the *h*_*s*_ value is 10^−4^~10^−5^ m. It is also consistent with the results which are shown in Figure 5 and the resolution of measurement system which is used in this study.

## 3. Discussion

In this work, we found that the contact angle was not affected by gravity, while the measured apparent contact angle was gravity-dependent. The measured apparent contact angle is not the real contact angle. Actually, it is the angle of inclination of the liquid-vapor interface far away from the solid surface. The contact angle cannot be measured by the optical method because it depends on the resolution of the measurement system. However, using a high-solution measurement system, one can obtained an approximation of the contact angle. This is of great significance for the accurate measurement of the contact angle and the development of a new contact angle measurement system. Furthermore, with the combination of theoretical derivation and experimental measurements, we obtained a new equation (Equation (12)), which can be used to calculate the apparent contact angle at different gravities based on the resolution of measurement system. This study can provide new horizons for solving the debate on whether gravity affects contact angle.

## 4. Materials and Methods

Purified water (18 M*Ω*·cm, obtained using a Millipore Milli-Q system), ethylene glycol (CP, purchased from Sinopharm Chemical Reagent Co. Ltd., China), and glycerol (CP, purchased from Sinopharm Chemical Reagent Co. Ltd., China) were chosen as probe liquids.

Silicon wafers (100 orientation, P/B doped, resistivity from 20 to 40 *Ω*·cm) were chosen as the substrate for the preparation of the probe surface, which were purchased from Zhejiang Li Jing Silicon Material Co. Ltd., China. Disks (4 inches) was cut into rectangular shapes of about 2 cm × 4 cm and then soaked in a freshly prepared mixture of 7 parts concentrated with sulfuric acid and 3 parts 30% hydrogen peroxide at 150°C for 30 min, rinsed with purified water, dried in a clean oven at 120°C for 1-2 h, and then allowed to cool to room temperature.

### 4.1. Preparation of DMDCS Surface

The dry silicon wafers were transferred to a flask containing 0.5 mL of DMDCS. To make sure there was no direct contact between the liquids and solid surfaces, silicon wafers were placed in an oven at 70°C for 3 days, and then rinsed sequentially with toluene, ethanol, ethanol-purified water (1 : 1), and purified water, and dried in a clean oven at 120°C for 1-2 h [42].

### 4.2. Preparation of PDMS Surface

The dry silicon wafers were transferred to a flask and wet with PDMS^2K^ or PDMS^9K^(purchased from Sinopharm Chemical Reagent Co. Ltd., China), placed in a clean oven at 100°C for 24 h, rinsed sequentially by copious toluene, acetone, and purified water, and dried in a clean oven at 120°C for 1-2 h [41].

### 4.3. Determination of Solid Surface Morphology

The surface morphology was studied using atomic force microscopy (PicoPlus AFM, manufactured by Modular Imaging, USA), and tapping mode was selected. The scanned domain was 10.0 *μ*m × 10.0 *μ*m. The roughness of the sample surfaces was analyzed from the AFM images with PicoView 1.12 Software (Agilent Technologies, USA).

### 4.4. Measurement of Contact Angle

The contact angles of liquids on solid surfaces were measured under different gravities generated by a long-arm centrifuge. Each measurement was carried out at a new location on the sample surface. The advancing/receding velocity of the three-phase contact line was 0.1-1.5 mm/min.

## Figures and Tables

**Table 1:** *h*_*s*_ values (*μ*m) of the solid-liquid contact system.

Gravity	Water on DMDCS	Ethylene glycol on DMDCS	Glycerol on DMDCS	Water on PDMS^2k^	Ethylene glycol on PDMS^2k^	Glycerol on PDMS^2k^	Water on PDMS^9k^	Ethylene glycol on PDMS^9k^	Glycerol on PDMS^9k^
1 G	22.6	13.3	13.2	22.2	18.2	12.9	9.5	29.0	4.8
2 G	22.6	12.5	30.4	19.4	23.7	26.3	30.8	28.9	4.8
3 G	20.4	12.5	46.5	29.5	19.7	42.4	22.6	25.0	4.8
4 G	25.3	11.6	45.6	22.6	17.7	49.1	32.9	39.7	6.6
5 G	32.2	14.1	45.2	31.8	30.6	49.2	31.9	48.6	8.6
6 G	30.2	13.2	57.9	38.9	31.8	47.2	30.0	50.1	23.2
7 G	40.3	14.9	57.8	39.7	30.8	52.1	35.0	52.1	22.8
8 G	38.2	21.3	56.8	48.2	37.4	58.4	41.1	54.1	22.7
Average	28.9 ± 2.7	14.2 ± 1.1	44.2 ± 5.5	31.5 ± 3.6	26.2 ± 2.6	42.2 ± 5.3	29.2 ± 3.4	40.9 ± 4.2	12.3 ± 3.1

## Data Availability

All data are available in the manuscript or supplementary materials.

## Supplementary Materials

Supplementary Materials Figure S1: variation of the contact angle with the three-phase contact radius under different gravities. (a)–(h) The contact angles versus time for water on a DMDCS surface under 1-8 G gravitational levels. (a′)– (h′) The three-phase contact radius versus time under 1-8 G gravity. Figure S2: schematic flowchart for calculation. (a) Schematic flowchart for adjusting the value of the length *h*_*s*_. (b) Young's contact angles (*θ*_Y_), solid surface tensions (*γ*_sv_), and solid-liquid interfacial tensions (*γ*_sl_) of solid-liquid contact systems. (c) The maximum height of the large sessile drop at equilibrium under different gravities. (2) Derivation of the relationship between gravity and the apparent contact angle. (3) Calculation of the value of the solid-liquid interfacial tension and solid surface tension.
