# Erratum to “The Possibility of Changing the Wettability of Material Surface by Adjusting Gravity”

**DOI:** 10.34133/2020/7646809

**Published:** 2020-03-20

**Authors:** Yong-Ming Liu, Zi-Qing Wu, Sheng Bao, Wei-Hong Guo, Da-Wei Li, Jin He, Xiang-Bin Zeng, Lin-Jun Huang, Qin-Qin Lu, Yun-Zhu Guo, Rui-Qing Chen, Ya-Jing Ye, Chen-Yan Zhang, Xu-Dong Deng, Da-Chuan Yin

**Affiliations:** ^1^Key Lab of Space Bioscience & Biotechnology, School of Life Sciences, Northwestern Polytechnical University, Xi'an, 710072 Shaanxi, China; ^2^School of Bioengineering, Sichuan University of Science and Engineering, Zigong, 643000 Sichuan, China; ^3^Shenzhen Research Institute of Northwestern Polytechnical University, Shenzhen, 518057 Guangdong, China

In the article titled “The Possibility of Changing the Wettability of Material Surface by Adjusting Gravity” [1], there were errors in Figure 2 which occurred during production. These errors are listed below, and the corrected version is shown as Figure 1:

In panel (b), “ACA of ethylene aglycol” should read “ACA of ethylene glycol.”

In panel (c), “The difference in contract angles at 1G and 8G” should read “The difference in contact angles at 1G and 8G.”

In panel (c), “ED: Ethylene on DMDCS” should read “ED: Ethylene glycol on DMDCS.”

In panel (c), “E2K; Ethylene on PDMS^2K^” should read “E2K; Ethylene glycol on PDMS^2K^.”

In panel (c), “E9K: Ethylene on PDMS^9K^” should read “E9K: Ethylene glycol on PDMS^9K^.”

In panel (c), the blue squares should represent “Δ*H*: The average contact angle hysteresis,” and the red circle should represent “Δ*θ*: The difference in contact angles at 1G and 8G.”

In the figure caption of Figure 2, the sentence “Comparison of Δ*θ* (the difference in contract angles at 1G and 8G) and Δ*H* (the average contact angle hysteresis)” should read “Comparison of Δ*θ* (the difference in contact angles at 1G and 8G) and Δ*H* (the average contact angle hysteresis).”

## Figures and Tables

**Figure 1:**
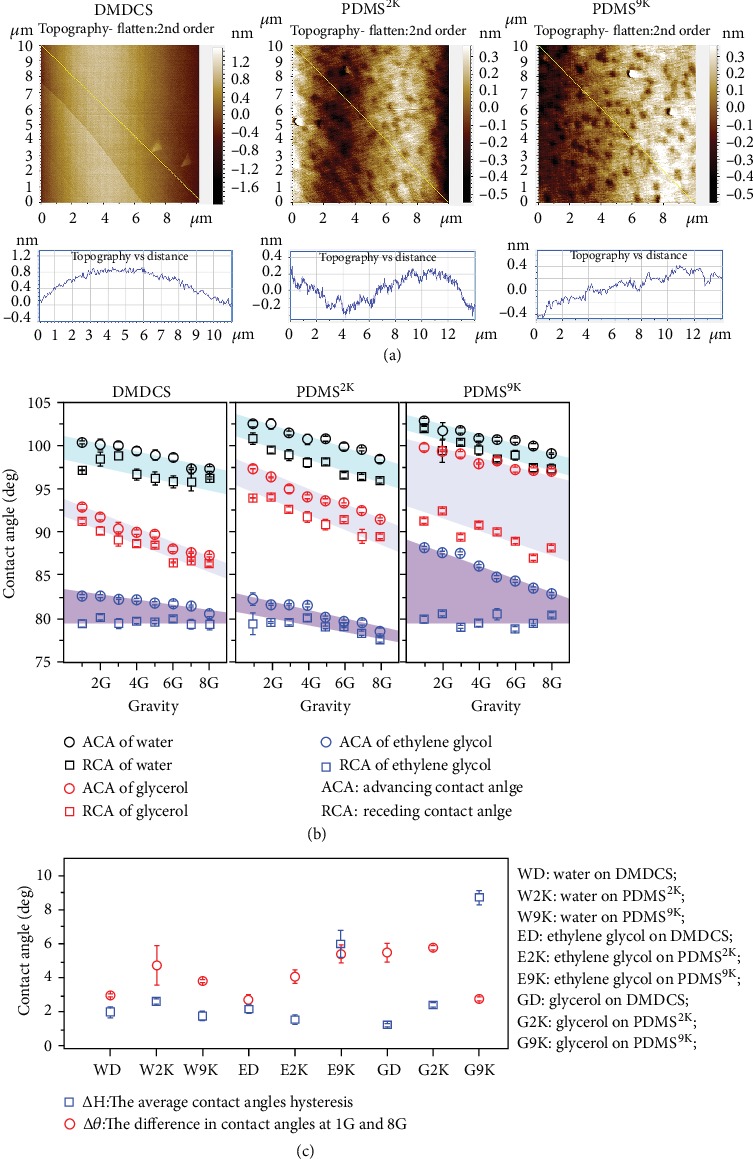
Apparent contact angles versus the gravity. (a) AFM images of the sample solid surfaces and their section analyses. The analyses show that the surfaces are very smooth, with roughness less than 1 nm. (b) The contact angle on solid surfaces at different gravities. The contact angles show the same tendency to decrease with increasing gravity. The contact angle hysteresis was smaller than 3°, except for the cases of ethylene glycol and glycerol on PDMS^9K^. (c) Comparison of Δ*θ* (the difference in contact angles at 1 G and 8 G) and Δ*H* (the average contact angle hysteresis). Independent *t* test was applied, *n* = 3, *P* < 0.05, *P* < 0.001, *P* < 0.001, *P* < 0.05, *P* < 0.001, and *P* < 0.001, for water on DMDCS, water on PDMS^9K^, ethylene glycol on PDMS^2K^, glycerol on DMDCS, glycerol on PDMS^2K^, and glycerol on PDMS^9K^, respectively. Five out of the nine solid-liquid contact systems showed significant larger Δ*θ* than Δ*H*, and three showed comparable results. The experimental results confirmed that the decrease in contact angle upon increasing gravity is caused by the gravity, not only by the contact angle hysteresis.
