# Supplementary material for: The Possibility of Changing the Wettability of Material Surface by Adjusting Gravity
Source: Research (Wash D C). 2020 Jan 27;2020:2640834. doi: 10.34133/2020/2640834 (PMC7007757; doi:10.34133/2020/2640834)
Supplement: Supplementary Materials — Figure S1: variation of the contact angle with the three-phase contact radius under different gravities. (a)–(h) The contact angles versus time for water on a DMDCS surface under 1-8 G gravitational levels. (a′)– (h′) The three-phase contact radius versus time under 1-8 G gravity. Figure S2: schematic flowchart for calculation. (a) Schematic flowchart for adjusting the value of the length hs. (b) Young's contact angles (θY), solid surface tensions (γsv), and solid-liquid interfacial tensions (γsl) of solid-liquid contact systems. (c) The maximum height of the large sessile drop at equilibrium under different gravities. (2) Derivation of the relationship between gravity and the apparent contact angle. (3) Calculation of the value of the solid-liquid interfacial tension and solid surface tension. [file 2640834.f1.docx]

Supplemental Material

The Possibility of Changing the Wettability of Material Surface by Adjusting Gravity

Yong-Ming Liu^1,2^,†, Zi-Qing Wu^1^,†, Sheng Bao^1^, Wei-Hong Guo^1^, Da-Wei Li^1^, Jin He^1^, Xiang-Bin Zeng^1^, Lin-Jun Huang^1^, Qin-Qin Lu^1^, Yun-Zhu Guo^1^, Rui-Qing Chen^1^, Ya-Jing Ye^1^, Chen-Yan Zhang^1^, Xu-Dong Deng^1^, and Da-Chuan Yin^1,3^*

^1^ Key Lab of Space Bioscience & Biotechnology, School of Life Sciences, Northwestern Polytechnical University, Xi’an 710072, Shaanxi, PR China.

^2^ School of Bioengineering, Sichuan University of Science and Engineering, Zigong 643000, Sichuan, PR China

^3^ Shenzhen Research Institute of Northwestern Polytechnical University, Shenzhen 518057, Guangdong, PR China

† These authors contributed equally

* Correspondence should be addressed to Da-Chuan Yin; yindc@nwpu.edu.cn

1. Supplementary Figures


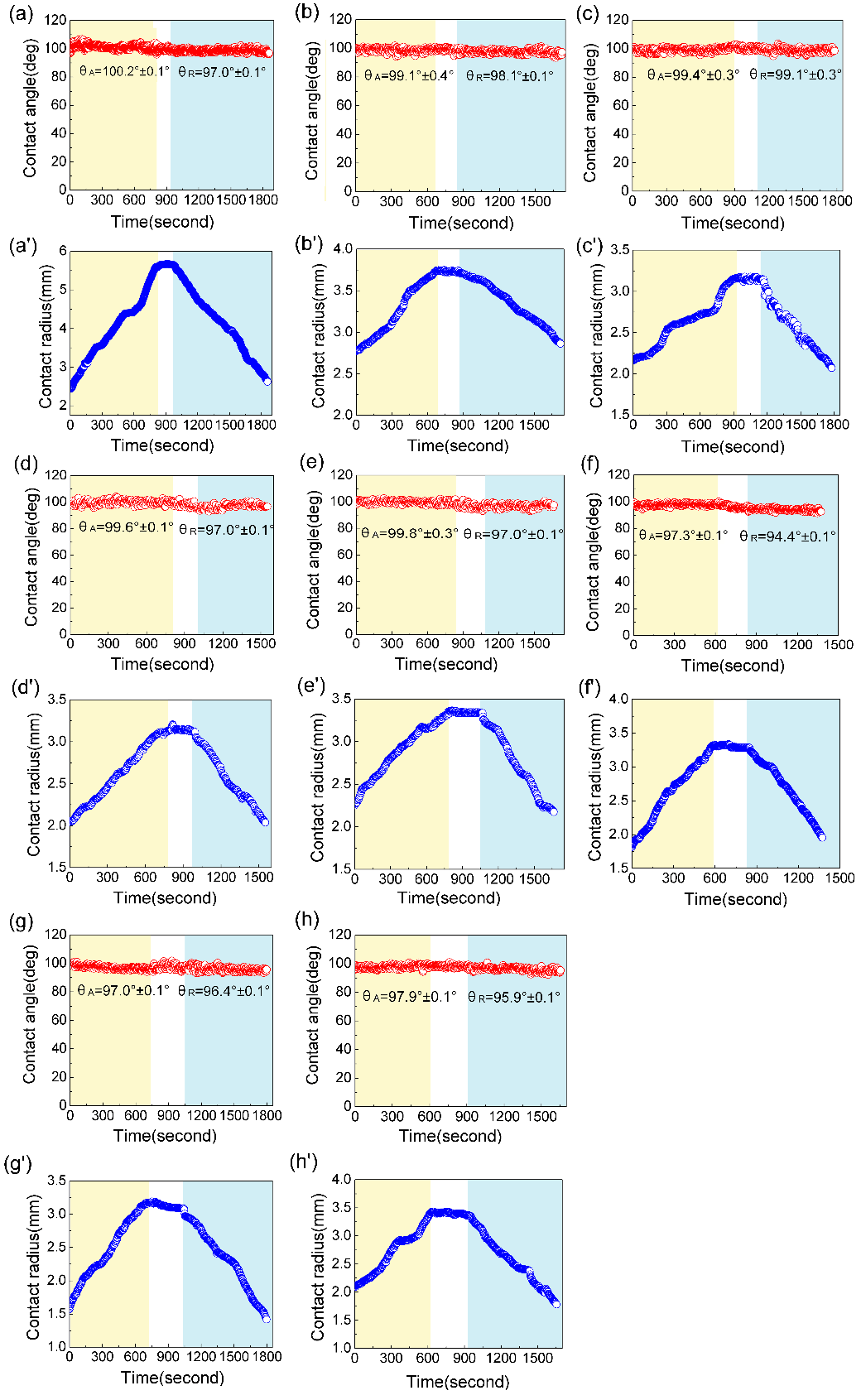


**Figure S1.** Variation of the contact angle with the three-phase contact radius under different gravities. (a)-(h), The contact angles versus time for water on a DMDCS surface under 1-8 G gravitational levels. (a')-(h'), The three-phases contact radius versus time under 1-8 G gravity.


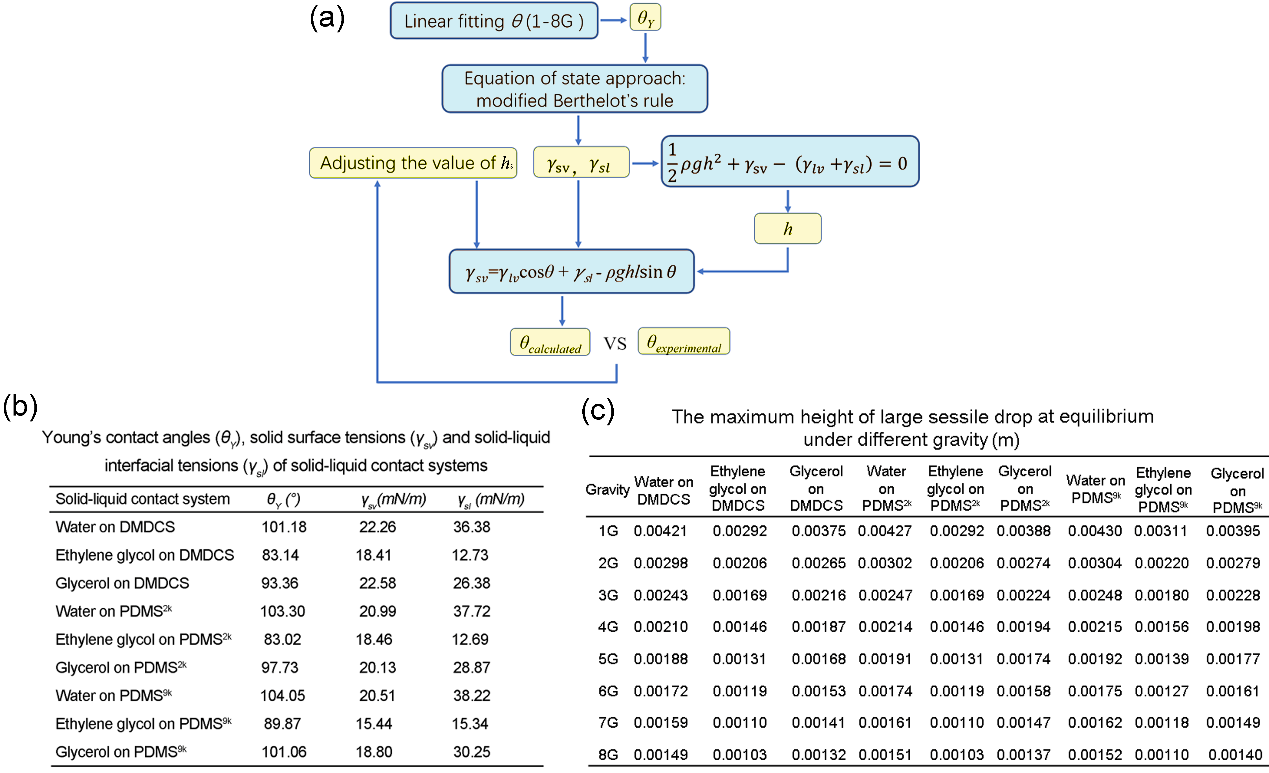


**Figure S2.** Schematic flowchart for calculation. (a) Schematic flowchart for adjusting the value of the length *h_s_.* (b) Young’s contact angles (*θ_Y_*), solid surface tensions (*γ_sv_*) and solid-liquid interfacial tensions (*γ_sl_*) of solid-liquid contact systems. (c) The maximum height of large sessile drop at equilibrium under different gravities.

2. Derivation of the relationship between gravity and apparent contact angle

For a large drop, we consider a small rectangular section (ABCD, see Fig. 4 in the main paper) of the liquid surface near the three-phase contact line, where CD is the three-phase contact line. The liquid-vapor interfacial tension forces pull the three edges of surface ABCD along the tangent direction perpendicular to the edges, and the solid-liquid and the solid-vapor interfacial tension forces *F_sl_* and *F_sv_* pull side CD along the horizontal direction. The pulling force *F_1_* on side AB along the tangent direction is

$F_{1}=\gamma_{lv}\cdot AB$, (S1)

where *γ_lv_* is the liquid-vapor interfacial tension and AB is the length of side AB. The horizontal component of *F_1_* is

$f_{1}=F_{1}\cdot\cos\theta=\gamma_{lv}\cdot AB\cdot\cos\theta$, (S2)

where *θ* is the apparent contact angle. Force *F_2_* acts on side BC, and force *F_3_* acts on side AD. These two forces counterbalance each other; thus, the net force in that direction is 0.

The forces *F_sl_* and *F_sv_* act on side CD (Figure 4 in main paper). Their directions are opposite along the horizontal direction. These two forces can be written as

$F_{sl}=\gamma_{sl}\cdot CD$, (S3)

and

$F_{sv}=\gamma_{sv}\cdot CD$. (S4)

If the sessile drop is in a gravity *g*, the surface ABCD will experience a hydrostatic pressure, resulting in a force *F_g_* that is perpendicular to the surface. The force *F_g_* can be written as:

$F_{g}=\rho g(h_{e}-\frac{1}{2}BC\sin\theta)\cdot AB\cdot BC$, (S5)

where *ρ* is the density of the liquid and *h_e_* is the height of the drop. The horizontal component of force *F_g_* is

$f_{g}=\rho g(h_{e}-\frac{1}{2}BC\sin\theta)\cdot AB\cdot BC\cdot\sin\theta$. (S6)

The sum of the forces in the horizontal direction must be zero:

$\begin{matrix} f_{1}+F_{sl}+F_{sv}+f_{g}=\gamma_{lv}\cdot AB\cdot\cos\theta+\gamma_{sl}\cdot CD-\gamma_{sv}\cdot CD \\ \begin{matrix} & & & & & \end{matrix}-\rho g(h_{e}-\frac{1}{2}BC\cdot\sin\theta)\cdot AB\cdot BC\cdot\sin\theta\\ \begin{matrix} \end{matrix}\begin{matrix} \end{matrix}\begin{matrix} \end{matrix}\begin{matrix} & & \end{matrix}\begin{matrix} & \end{matrix}=0 \end{matrix}$ (S7)

From Figure 4b, it can be seen that AB = CD. Equation (S7) can be rewritten as

$\gamma_{sv}=\gamma_{lv}\cdot\cos\theta+\gamma_{sl}-\rho gh_{e}(BC)\sin\theta\text{+}\frac{1}{2}\rho g(BC)^{2}\sin^{2}\theta$. (S8)

Since *l* is small, and sin*θ* is smaller than 1, the last item ($\frac{1}{2}\rho g(BC)^{2}\sin^{2}\theta$) is several orders of magnitude smaller than the third term ($\rho gh_{e}(BC)\sin\theta$). Hence Equation (S8) can be simplified as below:

$\gamma_{sv}=\gamma_{lv}\cdot\cos\theta+\gamma_{sl}-\rho gh_{e}(BC)\sin\theta$. (S9)

Let $BC\sin\theta$= *h_s_*, Equation (S9) can be rewritten:

$\gamma_{sv}=\gamma_{lv}\cdot\cos\theta+\gamma_{sl}-\rho gh_{e}h_{s}$. (S10)

3. Calculation of the value of solid-liquid interfacial tension and solid surface tension

The schematic flowchart of the calculation process for value of *l* is shown in Figure S3(a), where, Young’s contact angles *θ_Y_* for each solid-liquid contact system were obtained though the linear fitting of the data in Figure 2 in main paper; And the solid surface tension $\gamma_{sv}$, solid-liquid interfacial tension $\gamma_{sl}$ can be calculated using Equation (S10) and Equation (S11) respectively [1]. The results show in Figure S3(b).

(S10)

where, *β* is a constant, 1.247×10^-4^ (m^2^/mJ)^2^.

$\gamma_{sl}=\gamma_{lv}+\gamma_{sv}-2\sqrt{\gamma_{lv}\gamma_{sv}}e^{-\beta(\gamma_{lv}-\gamma_{sv})^{2}}$ (S11)

The height of the sessile drop under different gravities can be calculated by the using the Equation (S12) [2]. The results show in Figure S3(c).

$\frac{1}{2}\rho\text{gh}^{2}+\gamma_{sv}-(\gamma_{lv}+\gamma_{sl})=0$ (S12)

# 4. References

[1] D. Y. Kwok, A. W. Neumann, *Adv. Colloid. Interf. Sci.* **1999**, 81, 167-249.

[2] P. G. D. Gennes, F. Brochard-Wyart, D. Quéré. *Springer, Heidelberg*. **2004**, 36.
